# Design and implementation of a mobile app for the pharmacotherapeutic follow-up of patients diagnosed with immune-mediated inflammatory diseases: eMidCare

**DOI:** 10.3389/fimmu.2022.915578

**Published:** 2022-07-28

**Authors:** Rosa Romero-Jimenez, Vicente Escudero-Vilaplana, Esther Chamorro-de-Vega, Arantza Ais-Larisgoitia, Elena Lobato-Matilla, Beatriz Somoza-Fernández, Paula Ruiz-Briones, Carlos González, Ofelia Baniandrés, Luis Menchén, Carmen Lobo-Rodríguez, Ana Herranz, María Sanjurjo

**Affiliations:** ^1^ Pharmacy Department, Hospital General Universitario Gregorio Marañón, Madrid, Spain; ^2^ Instituto de Investigación Sanitaria Gregorio Marañón, Madrid, Spain; ^3^ Rheumatology Department, Hospital General Universitario Gregorio Marañón, Madrid, Spain; ^4^ Dermatology Department, Hospital General Universitario Gregorio Marañón, Madrid, Spain; ^5^ Gastroenterology Department, Hospital General Universitario Gregorio Marañón, Madrid, Spain; ^6^ Nursing Department, Hospital General Universitario Gregorio Marañón, Madrid, Spain

**Keywords:** communication, immune-mediated inflammatory disease, mHealth, mobile app, monitoring

## Abstract

**Background:**

Pharmacotherapeutic management of immune-mediated inflammatory diseases (IMID) has become more complex due to the development of new treatments, such as biological therapies. Mobile health, especially apps, can provide IMID patients with greater autonomy and facilitate communication with healthcare professionals. Our objective was to design and implement an app for remote monitoring and communication with IMID patients.

**Methods:**

A multidisciplinary group was created to design and develop an app for IMID patients in a tertiary hospital. The app functionalities were identified through a focus group with IMID patients and through an observational, descriptive study of available apps for IMID patients at App Store and Play Store platforms. Once the app was designed and developed, we offered the app to IMID patients who initiated a new biological therapy. The inclusion period was from December 2020 to August 2021. We performed an observational, longitudinal study to assess the app’s impact on medication safety, communication, satisfaction, and usability.

**Results:**

We designed an app (eMidCare^®^) with the following modules: My Medication, My Questionnaires, Adverse Events, Useful Information, Messages, and Patient Profile. A total of 85 patients were installed with the app. The median (range) follow-up time for app use was 123 (5-270) days. In the My Medication module, 100% of patients registered their biological therapy and 25.9% also used this module to record each dose of medication administered. A total of 82 adverse events (AEs) were registered. Thirty-two percent of the patients registered at least 1 AE. The most frequent AEs were fatigue, injection site reaction, headache, and nausea. Fifty-two percent of patients used the Messages module to communicate with healthcare professionals. The most frequent messages concerned doubts about managing AEs (26.2%) and drug interactions (18.9%). The satisfaction survey yielded a median (range) score of 9.1 (7-10) out of 10.

**Conclusions:**

We developed an app, eMidCare^®^, which reminds patients to take their medication, enables them to record AEs, and helps them communicate with healthcare professionals. Approximately one-third of the patients registered the administration of the biological therapies and registered at least 1 AE. The most used and most satisfactory functionality was communication with health professionals.

## 1 Introduction

The management of several immune-mediated inflammatory diseases (IMID) has changed dramatically owing to the development of biological therapies (1). These drugs resolve inflammatory processes quickly and effectively, thus improving prognosis. However, biological therapies continue to be associated with drug-related problems, such as adverse events and poor adherence (2). Furthermore, pharmacotherapeutic management of IMID has become more complex, and patients are now responsible for the correct administration of their treatment (3).

The quality of life of IMID patients is often affected by physical and psychological burdens (4–6). Therefore, it is essential to assess patient-reported outcomes (PROs) to complete clinical records and guarantee adequate care (4). Patient involvement is a crucial element in national health systems because of its impact on health outcomes and costs. Healthcare professionals should encourage the empowerment of IMID patients.

Information and communications technology, specifically mobile health (mHealth), can provide IMID patients with greater autonomy, facilitate communication with healthcare professionals, and increase the quality of patient care (7). Remote patient monitoring could lead to early identification of adverse events, early intervention for patients requiring their treatment to be adjusted, and reduced emergency room visits (7). IMID patients are younger than patients with other chronic diseases and, therefore, more familiar with these technologies (1). In addition, they are very eager to use mHealth to better understand their diseases (8). Apps represent an excellent opportunity to improve self-management of care and enable healthcare professionals to improve monitoring and patient care.

Our objective was to design and implement an app for self-management of disease and thus promote home monitoring and communication with IMID patients. We also assessed the usability of and satisfaction with the app.

## 2 Material and methods

### 2.1 App design

A multidisciplinary working group comprising pharmacists, dermatologists, rheumatologists, gastroenterologists, and nurses was created to design and develop an app for IMID patients in a tertiary hospital. The functionalities of the app were based on the following: patients’ healthcare needs and their interest in the use of apps to manage their disease; and functionalities and uncertainties of currently available IMID apps.

1) Patients’ healthcare needs and their interest in the use of apps to manage their disease

We used the focus group methodology with IMID patients. A meeting was organized with 7 patients (2 diagnosed with psoriasis, 2 with psoriatic arthritis, and 3 with ankylosing spondylitis). The median age was 47.7 (SD= 10.3) years, and all patients were receiving biological therapy.

2) Functionalities and uncertainties characteristic of currently available IMID apps

We conducted a descriptive study of all apps for IMID patients to identify areas that could be improved with our new app. We searched the App Store (iOS) and Play Store (Android). The search terms used were “ankylosing spondylitis”, “Crohn’s disease”, “hidradenitis suppurativa”, “IBD”, “inflammatory bowel disease”, “immune-mediated inflammatory diseases”, “immune-mediated inflammatory disorders”, “juvenile idiopathic arthritis”, “psoriasis”, “psoriatic arthritis”, “rheumatoid arthritis”, and “ulcerative colitis”. The variables analyzed were app name, type of IMID, platform (Android or iOS), country of origin, language, category of the app, functionalities, date of the last update, downloads, and author affiliation.

### 2.2 App implementation

The app was designed and developed considering the points mentioned above. In December 2020, we started offering the app to all IMID patients who initiated a new biological therapy. We performed an observational, longitudinal study of patients diagnosed with an IMID and followed using the app eMidCare to assess the tool’s impact on medication safety, communication, satisfaction, and usability. The inclusion period was from December 2020 to August 2021. The inclusion criteria were age ≥ 18 years, diagnosis of an IMID (ankylosing spondylitis, hidradenitis suppurativa, inflammatory bowel disease, juvenile idiopathic arthritis, psoriatic arthritis, psoriasis, and rheumatoid arthritis), and ownership of a Smartphone. Patients with language barriers were excluded. The study was approved by the Hospital Clinical Research Ethics Committee (code MSS-APP-2019). All patients signed an informed consent document.

Patients were offered the app by the pharmacist in the outpatient pharmaceutical care clinic. If the patient agreed to participate, the pharmacist registered the patient on the Content Management System (CMS) website, along with the delivery of the biological therapy and the usual information provided in the pharmaceutical care clinic. Once the patient downloaded the app, the pharmacist explained how to use it. Healthcare professionals then followed the patients up through the CMS. Health professionals can modify the contents of the app through the CMS (for example, they can add new PROs, change the recommendations for adverse events, or modify useful information when necessary).

Medication safety was evaluated through a descriptive analysis of the patient’s recording of adverse events (AE) using the app, the percentage of patients who registered an AE, type of AE, time of onset, and severity according to CTCAE (9).

Communication was evaluated through the messages sent through the app, the percentage of patients who used this module, whole messages, messages per patient, and type of messages.

Usability was evaluated using Google Analytics for Firebase^®^.

Satisfaction with the app was assessed through a specific survey on the app. The survey included items on usability, ease of communication with healthcare professionals, utility for the management of treatment, and grade of recommendation (**Supplemental Material**). The survey was delivered between 1 and 2 months after the app was installed.

## 3 Results

### 3.1 App design

1) Patients’ healthcare needs and their interest in the use of apps to manage their disease

The patients in the focus group highlighted the following issues:

General healthcare needs:

- Lack of information on diseases, lifestyles, and treatments
- Lack of knowledge on the part of healthcare professionals (e.g., general practitioners and surgeons) with respect to the disease and treatments
- Limited mobility owing to the consequences of the diseases
- Reduced possibility of communication with the medical specialist (only from Monday to Friday from 9 am to 2 pm)
- Considerable interest in using information and communication technologies, especially apps, to search for information about health

Desired health app functionalities:

- General information about the disease and treatments: symptoms, compatible medication, adverse effects, feeding, surgery, physical activity, vaccines, drug conservation, travel
- Specific information about AE management (e.g., when to go to the emergency room)
- Medication alarms
- Ability to register the injection site in the case of subcutaneous medication
- Diary to record AEs and symptoms
- Ability to take photographs of the skin and send them to specialists
- Questionnaires to assess and monitor physical and emotional states (patient-reported outcomes [PROs])
- Direct contact with health professionals to clarify doubts

Key elements of app design: Patients need information about their disease and treatments and must be able to communicate with healthcare professionals. They highlight different functionalities of the apps that will help them to manage their disease and should be incorporated in our app.

2) Functionalities and uncertainties characteristic of currently available IMID apps

We analyzed 243 apps (37.0% on Android, 27.2% on iOS, and 35.8% on both platforms). The main functionalities were as follows: general information about lifestyles, nutrition, and exercise (135/243, 55.6%), specific information about the disease or treatment (102/243, 42.0%) (although only 6 apps offered information specifically about biological therapies), recording adverse events (51/243, apps 21.0%), agenda/calendar (44/243, 18.1%), medication reminder (41/243, 16.9%), and recording of patient-reported outcomes (41/243, 16.9%). Healthcare professionals were involved in the development of 100 apps (41.1%). Only 110/243 apps (44.5%) had been updated in the previous year. The mean time between the date of the analysis and the date of the last update was 18.5 months (SD= 19.3).

Key points of app design: Functionalities and reliability were very heterogeneous. While most apps offer information, although information on biologic therapies is very scarce. Few apps allow patient interaction, such as recording AEs, setting alarms for medication, or recording PROs. Our app should have these functionalities under the supervision of healthcare professionals as a guarantee of reliability.

The content of the app was defined based on the above key points and the experience of the multidisciplinary group. The computer company Enredart^®^, which specializes in developing and implementing health apps, was contracted for development. A document was drawn up with the design and functional scope of the app, the support service, and the implementation plan. We decided to call the app eMidCare. The app was structured into 6 modules:

1) **My Medication**: This module enables the patient to record the medicines he/she is taking (biological therapies and concomitant medicines), with details of the type of intake, dose, unit of measurement, frequency, and quantity. An icon for biological therapies makes it possible to obtain more detailed information about each drug. The medicines can be included in the app by patients or by healthcare professionals. Patients can receive reminders when they have to administer the medicines. They can also record when they administer each medication to monitor adherence (e.g. at the time of administration, 10 minutes before, half an hour before…). In the case of subcutaneous biological therapies, the patients may also register the injection site.

2) **My Questionnaires**: Healthcare professionals can upload PRO measures (PROMs) in the app and define the frequency and the PROMs for each IMID (**Table 1**). The PROMs (questionnaires) are programmed to be sent to the patients at the start of treatment, after 3 months, and then every 6 months, except for the Morisky-Green test, which is sent every 3 months. However, this frequency can be modified in the web interface (CMS). The healthcare professionals can add new questionnaires, modify existing ones, and change the sending frequency. Patients receive a reminder when a new questionnaire is available.

**Table 1:** Patient-reported outcomes assessed by the app.

PRO	PROM	Type of IMID						
		Ankylosing spondylitis	Hidradenitis suppurativa	Inflammatory bowel disease	Juvenile idiopathic arthritis	Psoriatic arthritis	Psoriasis	Rheumatoid arthritis
Quality of life	ASQoL	X						
	BASDAI	X				X		
	BASFI	X				X		
	DLQI		X				X	
	EQ-5D-5L	X	X	X	X	X	X	X
	IBDQ-9			X				
	PsAQoL					X		
	QoL-RA							X
Functional capacity	HAQ	X			X	X		X
Anxiety and depression	HAD	X	X	X	X	X	X	X
Adherence	Morisky-Green	X	X	X	X	X	X	X
Work productivity and activity impairment	WPAI	X	X	X	X	X	X	X

ASQoL, Ankylosing Spondylitis Quality of Life; BASDAI, Bath Ankylosing Spondylitis Disease Activity Index; BASFI, Bath Ankylosing Spondylitis Functional Index; DLQI, Dermatology Life Quality Index; EQ-5D-5L, EuroQol 5-dimension 5-level; HAQ, Health Assessment Questionnaire; IMID, Immune-mediated inflammatory diseases; HAD, Hospital Anxiety and Depression; IBDQ-9, Inflammatory Bowel Disease Questionnaire; PRO, Patient-reported outcome; PROM, Patient-reported outcome measure; PsAQoL, Psoriatic Arthritis Quality of Life; QoL-RA, Quality of Life-Rheumatoid Arthritis; WPAI, Work Productivity and Activity Impairment.

**3) Adverse Events**: Patients can register the adverse events they experience, and the app offers various recommendations on the type of AE and its severity. The system acts based on a defined decision tree (**Supplemental material**), which guides the patient through a series of questions and classifies the severity of the AE (grades 1-4) (9). Then, depending on the severity, the app gives automatic recommendations on appropriate management. For example, when severity is low (grade 1), the app displays a brochure with information on the prevention and management of each AE. The decision tree is implemented in the CMS. The healthcare professionals can add new AEs to the decision tree or change the questions and recommendations provided by the app thanks to this tree.

The multidisciplinary group defined this decision tree for the most frequent AEs: diarrhea, fatigue, fever, headache, nausea, injection site reaction, vomiting, and other (recorded as free text).

**4) Useful Information**: This module contains recommendations and preventive information on various areas of treatment (especially on biological therapies) and IMIDs. The information is structured as Frequently Asked Questions, Adverse Events, General Information (recommendations on nutrition, conservation and stability of drugs, travel recommendations, and information about the hospital), web links of interest, and a tutorial about the app. All the information was adapted to a language that patients can understand and is periodically updated.

**5) Messages**: This module allows bidirectional communication between patients and healthcare professionals in real time.

**6) Patient Profile**: This module contains information about the patient: sex, date of birth, weight, smoking habits, type of IMID, date of diagnosis, and secondary IMID and date of diagnosis (if applicable). The Patient profile module contains a section entitled “My achievements”, where patients can obtain “trophies” and “medals” depending on their adherence and the registration of the questionnaires.

**Figure 1:**
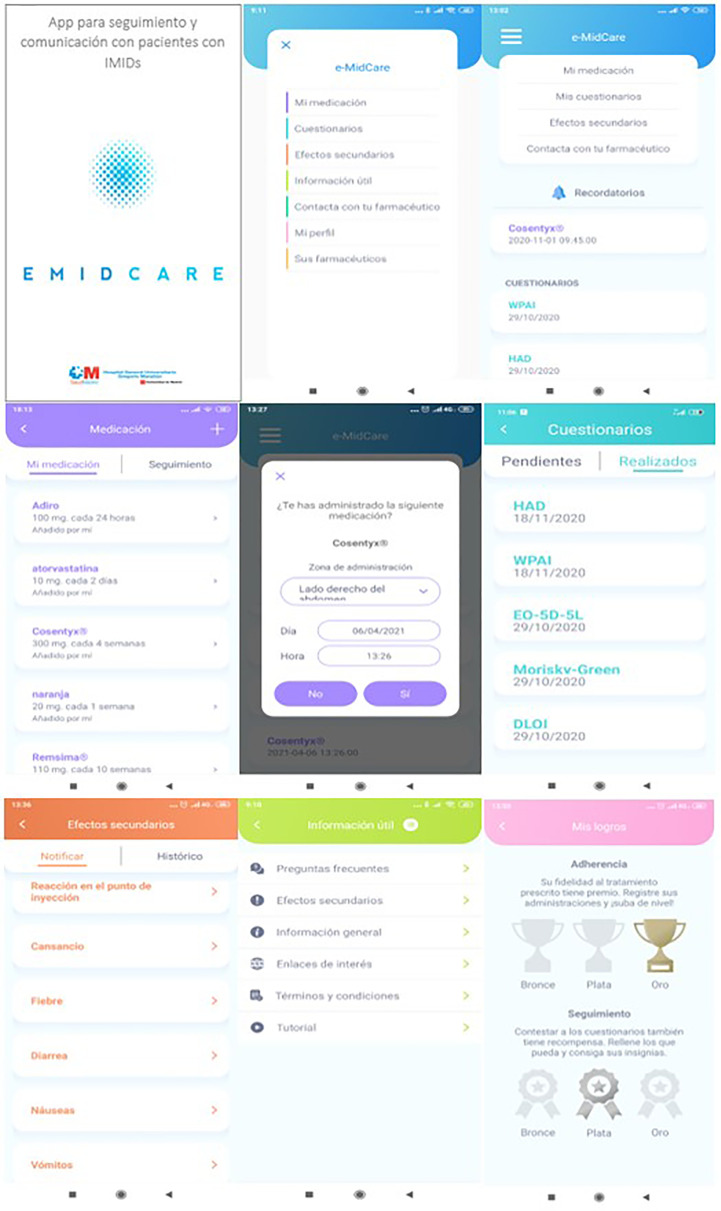
Functional diagram of eMidCare^®^ app screens.

**Figure 1** shows different eMidCare app screens.

#### 3.1.1 Privacy and security of the eMidCare platform

All the data registered in the app are recorded automatically in the web interface (CMS), which enables the healthcare professional to monitor patients in real time. The app also sends an automatic email to healthcare professionals when patients add a new medication, register an AE, or send a message. All the registered information can be exported to Excel^®^ for analysis.

All the data contained in the eMidCare platform is considered confidential and has the highest level of protection according to General Data Protection Regulation (GDPR).

The app does not store data locally, so it is not possible to access it once the user is deactivated from the program. The data travels from the CMS on the server to the app through encrypted webservices. The platform is located in a hosting network at the company Gigas. The backup solution of the hosted data through advanced backup is implemented in virtual appliances R1Soft.

Other characteristics concerning privacy and security are as follows:

-The CMS and webservices will be under the Secure Sockets Layer (SSL) certificate.
-Email and password of the user will be encrypted
-The security password level is displayed to the user with a bar based on 3 factors: uppercase, lowercase, and numbers. The eMidCare prototypes for iOS and Android and the CMS were tested by end-users in a staging environment (alpha testing) and then in a production environment (beta testing). After 5 preliminary versions, the definitive version of the app was finally uploaded to the market in December 2020.

### 3.2 App implementation

**Figure 2:**
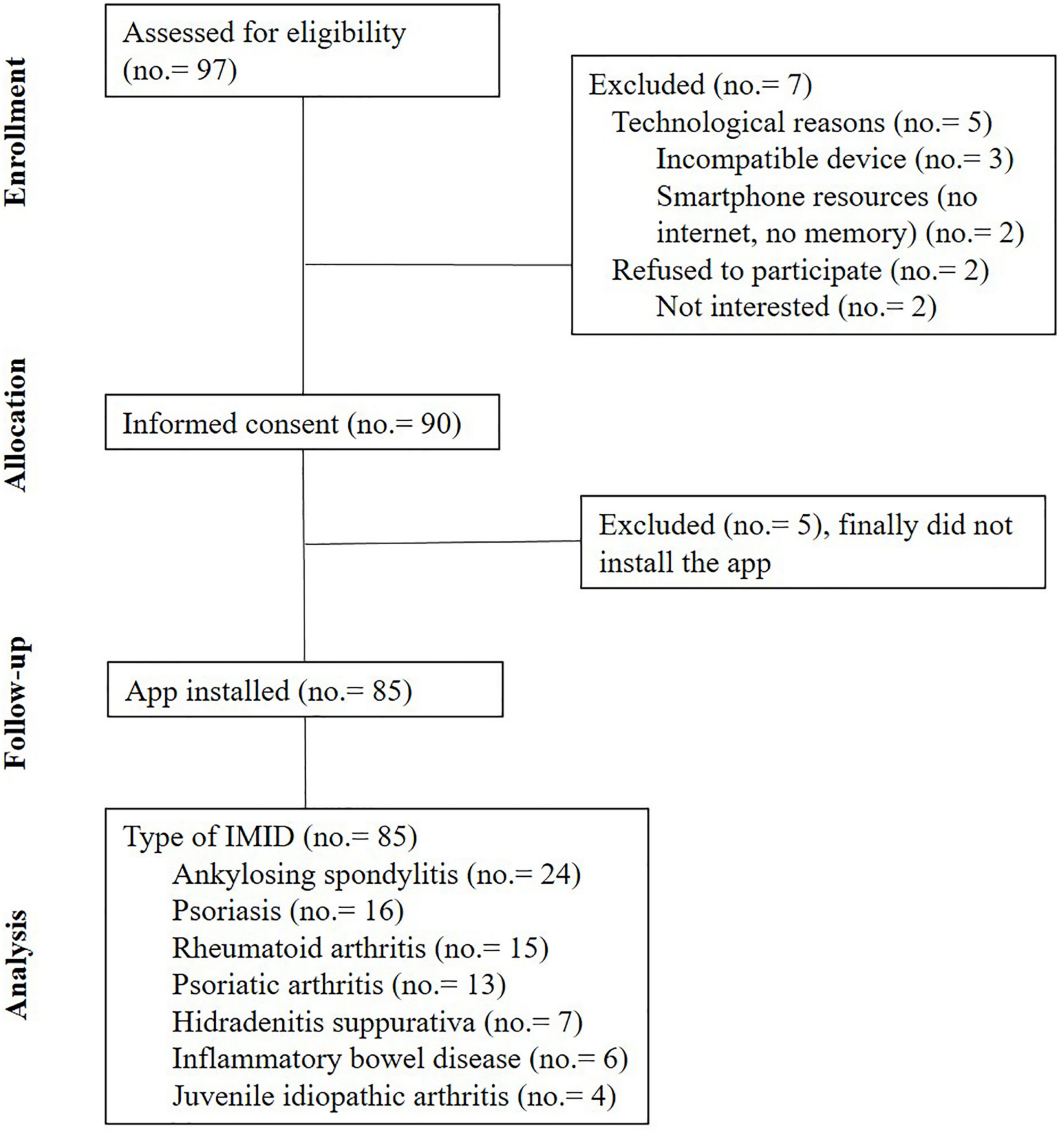
Patient inclusion diagram.

The app eMidCare was offered to 97 patients during the inclusion period, although 85 patients finally installed the app and were analyzed (**Figure 2**). A total of 53/85 patients (62.4%) were female. The median age (range) was 48.3 (18.1-79.4) years, and the median (range) weight was 70 (40-132) kg. **Table 2** describes the type of immune-mediated inflammatory diseases and the biological therapies used.

**Table 2:** Type of immune-mediated inflammatory diseases and biological therapy used.

CHARACTERISTIC		N (%)
Type of immune-mediated inflammatory	Ankylosing spondylitis	24 (28.2%)
	Psoriasis	16 (18.2%)
	Rheumatoid arthritis	15 (17.6%)
	Psoriatic arthritis	13 (15.3%)
	Hidradenitis suppurativa	7 (8.2%)
	Inflammatory bowel disease	6 (7.1%)
	Juvenile idiopathic arthritis	4 (4.7%)
Treatment	Adalimumab	38 (44.7%)
	Golimumab	7 (8.2%)
	Secukinumab	7 (8.2%)
	Etanercept	7 (8.2%)
	Guselkumab	5 (8.2%)
	Certolizumab	4 (4.7%)
	Apremilast	3 (3.5%)
	Tofacitinib	3 (3.5%)
	Ustekinumab	3 (3.5%)
	Baricitinib	2 (2.4%)
	Tildrakizumab	2 (2.4%)
	Abatacept	1 (1.2%)
	Ixekizumab	1 (1.2%)
	Risankizumab	1 (1.2%)
	Tocilizumab	1 (1.2%)
Biological therapy–naive	Yes	46 (54.1%)
	No	39 (45.9%)

In the My Medication module, all patients (85/85) registered their biological therapy (name, start date of treatment, dose, and administration frequency). Twenty-two (25.9%) also used this module to record each dose of medication administered. The median (range) adherence was 100% (50-100); adherence was >90% in 86.4% (19/22).

#### 3.2.1 Medication safety

A total of 82 AEs were registered, and 31.8% (27/85) of patients registered at least 1 AE. The median (range) number of AEs per patient was 1 (1-17). The frequency and severity of AEs were as follows: fatigue, 26.8% (22/82; 9.1% [2/22] grade 1, 77.3% [17/22] grade 2, and 13.6% [3/22] grade 3); injection site reaction, 25.6% (21/82; 81.0% [17/21] grade 1 and 19.0% [4/21] grade 2); headache, 15.9% (13/82; 38.5% [5/13] grade 1, 38.5% [5/13] grade 2, and 23.1% [3/13] grade 3); nausea, 8.5% (7/82; 42.9% [3/7] grade 1, 42.9% [3/7] grade 2, and 14.3% [1/7] grade 3); diarrhea, 4.9% (4/82; 100% [4/4] grade 1); upper respiratory infection, 3.7% (3/82; 100% [3/3] grade 1); and other AEs, 14.6% (12/82; 75.0% [9/12] grade 1 and 25.0% [3/12] grade 2). There were no statistically significant differences in the recording of AEs between biological-naïve patients and biological-experienced patients (34.7% vs. 28.2%, respectively, *p*>.05). The median (range) time to the first AE was 16 (0-95) days.

#### 3.2.2 Patient communication

Fifty-two percent of patients used the Messages module to communicate with healthcare professionals during the follow-up period. A total of 321 messages were sent (164 from patients and 157 from healthcare professionals). The most frequent messages concerned doubts about managing AEs and drug interactions (**Table 3**). A total of 56/164 (33.5%) messages were sent outside working hours.

**Table 3:** Type of message sent by patients through eMidCare^®^.

Message Classification	N (%)
Management of adverse events or symptoms	43 (26.2)
Drug interactions	31 (18.9)
Problems or suggestions related to the app	30 (18.3)
Acknowledgment of the pharmaceutical care received	20 (12.2)
Medication delivery and hospital logistics	13 (7.9)
Dosage and administration	12 (7.3)
General information about treatment	6 (3.7)
Storage and transportation of medication	5 (3.0)
Other	4 (2.4)
**Total**	**164**

#### 3.2.3 Patient satisfaction

**Figure 3:**
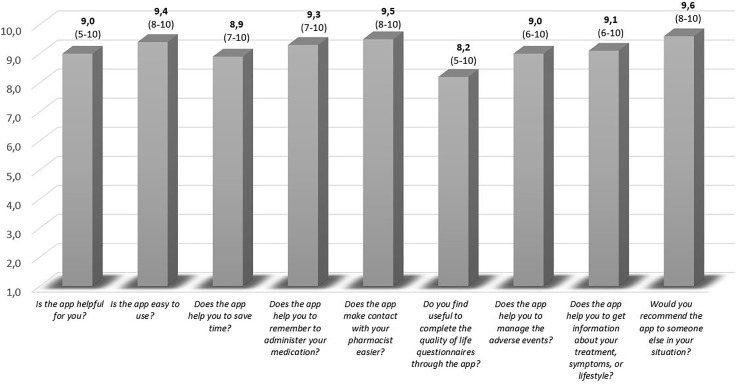
Patient satisfaction with eMidCare^®^. The score is expressed as the median (range).

The satisfaction survey yielded a median (range) score of 9.1 (7-10) out of 10. **Figure 3** describes the score for the various functionalities.

#### 3.2.4 Usability

The median (range) follow-up time for app use was 123 (5-270) days. The app sections that patients browsed for the longest time were Messages (21.9%), Start screen (20.9%), My questionnaires (20.4%), My medication (8.8%), Adverse events (7.1%), and reminders (5.7%). The median time per patient in each session with the app was 3.7 minutes.

The patients interacted an average of 1.4 times with the app each week. **Table 4** shows the app retention rate during the follow-up period (**Table 4**).

**Table 4:** App retention rate during the 9-month follow-up period.

	Months of follow-up								
	Month 1	Month 2	Month 3	Month 4	Month 5	Month 6	Month 7	Month 8	Month 9
December	100%	75%	75%	66%	50%	66%	50%	66%	50%
January	100%	75%	50%	75%	50%	66%	50%	75%	
February	100%	75%	75%	50%	75%	50%	75%		
March	100%	75%	75%	50%	66%	75%			
April	100%	75%	50%	50%	75%				
May	100%	75%	75%	85%					
June	100%	75%	75%						
July	100%	75%							
August	100%								

These data are expressed as the percentage of patients who interacted with the app at least once a week.

## 4 Discussion

### 4.1 Principal findings and comparison with previous studies

We report on the design and successful implementation of the app eMidCare for the home follow-up of IMID patients undergoing treatment with biological therapies. We assessed the app impact in 85 IMID patients, with characteristics similar to those of patients treated with biologic therapies. Currently available apps for IMID patients are considerably heterogeneous with respect to functionalities and reliability. A recent review of similar apps for Android and iOS has shown that more than half of IMID-related apps are oriented toward offering information (10). Nevertheless, a few apps allow for active patient participation (e.g., recording AEs, managing medication, and communicating with healthcare professionals). However, active use of apps should be encouraged for several reasons, namely, to promote empowerment and self-care and monitor these continuously in real-time and to obtain more information for decision-making by health professionals. Patients’ active participation in their disease care has been associated with improved health adherence, medication safety, and quality of life (11).

Concerning reliability, only 15-42% of IMID patient apps have been developed by professionals, and approximately the half of the apps have not been updated recently (10, 12–14). This limitation generates considerable doubt among professionals when recommending apps to patients. Therefore, we believe that an app designed by healthcare professionals whose impact is evaluated in a controlled environment, such as a hospital, guarantees reliability.

eMidCare was designed based on the experience of the multidisciplinary team and on patients’ needs. Few IMID-related apps have taken patients into account in their design. The 6 modules defined in eMidCare—My Medication, My Questionnaires, Adverse Events, Useful Information, Messages, and Patient Profile—meet the requirements for achieving our ultimate goal of improving medication safety, adherence, and communication with patients.

#### 4.1.1 My medication

Medication recording and reminders were the first functionalities incorporated into general health apps that enable patients to manage their pharmacotherapy. All patients included in the study recorded the biological therapy they were receiving. Consequently, they obtained specific information about each medication. In the market, there are very few apps with this functionality specific for this kind of therapies, only 5 out of 243 (2%) apps for IMID patients analyzed in a recent review had specific information on biologic therapies (10). Two of these apps (Remsimate^®^, MySIMPONI^®^) were oriented solely towards biologic therapy (infliximab and golimumab, respectively). However, this type of app should enable patients to record their complete treatment regimen, including monitoring of drug-drug interactions and adherence (13, 15, 16).

Apps are the preferred method for reminding patients to take their medication regularly (8), and numerous studies have shown that apps can improve adherence to treatment (13, 16–18). Alerts and reminders are the features that most frequently improve adherence. More than a quarter of our patients used this module to record each dose of medication administered. This could have influenced the high adherence observed (the median adherence was 100% and 86.4% of patients had an adherence >90%). Encouraging adherence is especially important in patients treated with biological therapies, since these medicines are not administered daily and it is easier to forget them. However, Romero-Jimenez et al. observed that only 16.9% of IMID-related apps enabled patients to record their medication and that only 11.1% enabled them to monitor adherence (10).

The Spanish Society of Hospital Pharmacy’s Rheumatoid Arthritis Guide references apps that have been used to improve adherence in IMID patients (RecuerdaMed^®^, MediSafe^®^, MyTherapy^®^, Dosecast^®^, Care4Today^®^) (19). However, none contain functionalities or information about IMID, but rather comprise general apps that aim only to remind patients to administer medication.

#### 4.1.2 My questionnaires

IMIDs have a high impact on patients’ quality of life (20). PROs enable the patient to provide subjective feedback on their disease, thus empowering the patient and enhancing patient-centred medicine (21). Monitoring PROs improves the follow-up of IMID patients and aids in decision-making, which improves health outcomes (22).

The use of apps can enhance recording and tracking of PROs’ by reducing limitations such as resource consumption, lack of time, and difficulties recording in real time and continuously (19). Riel et al. described different innovative electronic tools to monitor the management of arthritis (7). Sanoia^®^ is an online interactive electronic e-health platform that enables self-assessment and self-monitoring in rheumatoid arthritis (23). iMonitor^®^ is a software application that enables patients with rheumatoid arthritis, psoriatic arthritis, and ankylosing spondylitis to report information about their disease state. The physician can review PROs and receive alerts when established thresholds are not met or if PROs are not completed on time. GoTreatIT Rheuma^®^ is a software application that collects and displays data related to disease activity, health status, and quality of life. Despite these experiences, PRO recording is a rare functionality in apps for IMID patients. Romero-Jimenez et al. observed that only 11.5% of the apps for IMID patients enable recording of PROs (10). Another advantage of eMidCare is its versatility, which enables new PROMs to be incorporated. Healthcare professionals can easily incorporate PROMs directly into the CMS.

IMIDs can also have an impact on patients’ work activity. A cross-sectional online survey of spondyloarthritis patients from 13 European countries concluded that spondyloarthritis substantially impacts the working life of two-thirds of the actively employed population (24). Thus, we included the Work Productivity and Activity Impairment (WPAI) questionnaire in eMidCare.

#### 4.1.3 Adverse events

New biological therapies are becoming increasingly safe (1), although they may produce AEs that diminish their effectiveness and patients’ quality of life (25). Consequently, adequate pharmacovigilance is essential for prevention and management of AEs associated with this type of drug. Almost one-third of our patients reported at least one AE. Hence the important role of eMidCare in the safety of these treatments. Besides, patients took a median of 16 days to register their first AE, thus enabling us to identify them before the first follow-up consultation with a specialist. This early detection may have contributed to the early management of adverse effects, hence only 8.5% of the AE’s recorded were graded ≥ 3. For example, the proportion of patients who discontinued treatment with adalimumab in pivotal studies was 5.9% (25). A similar experience in cancer patients helped to reduce the number of AEs and their severity (15). Using a decision tree to manage AEs helped to reduce visits to the emergency room. However, this functionality is not standard in apps for IMID patients. Romero-Jimenez et al. observed that only 21.0% of the apps for IMID patients made it possible to record symptoms or AEs (10), although the authors did not state whether these apps offered individualized recommendations. We were unable to find apps for IMID patients that enable self-management of AEs through automatic recommendations *via* a decision tree.

#### 4.1.4 Useful information

Atreja et al. reported that IMID patients feel insufficiently informed about their illness and treatment (26), as our focus group participants highlighted.

Approximately 60% of the apps available for IMID patients offer information about the disease, treatments, and lifestyle (10), thus making it the most frequent functionality of these types of apps. Besides, information on medication was the feature most desired by a cohort of 193 patients with rheumatic disease (8). Mollard et al. observed that 25% of apps available for rheumatoid arthritis were exclusively for patient education (27). One of the advantages of eMidCare is that it includes specific information on biological therapies, which is rare in these apps (22).

Doubts about the reliability of the apps’ authorship mean that the information provided should be used with caution. Even more important is the timing of updates, especially in a field such as IMIDs and biological therapies, where new scientific evidence is constantly appearing. The lack of specific legislation means that there are no guarantees with respect to the information obtained (28, 29). However, information that points to an app’s reliability includes authorship, date of update, whether the information is referenced, number of downloads, ratings, comments on the stores, or even if it has a quality seal (30).

#### 4.1.5 Messages

In the focus group, we conducted prior to the development of the app, the most important point was to improve communication with healthcare professionals. This need has been confirmed with the use of eMidCare, as it has also been the most used functionality. The Messages module was used to communicate with the healthcare professional by 52% of patients. It was also the module most valued by patients, with a score of 9.5 out of 10. Messages was the most frequently accessed module, thus confirming the importance of these technologies in terms of communication. In addition, it was the most frequently used module in terms of time spent (21.9%).

We believe this app has greatly improved communication with our patients over the traditional model of care. The possibility of daily communication with health professionals reassures patients and gives them confidence. Thanks to eMidCare, we are helping patients who experience more difficulties contacting the health system and feel more helpless, since 33.5% of the queries answered through e-MidCare were made after 3:00 pm or at weekends. This communication proved to be worthwhile, since we likely avoided unscheduled visits.

In a French, multicenter, clinical trial, the interactive Sanoia^®^ e-health platform was compared with traditional follow-up for 12 months (23). The authors observed a statistically significant improvement in patients’ perception of the quality of communication between IMID patients and their physicians.

#### 4.1.6 Patient profile

The most innovative functionality of this module was gamification. The patient receives different awards according to the percentage of registration of medications and PROs. Gamification is a specific mHealth technique that favors behavioral change and increases users’ engagement. Haase et al. reported that gamification encourages adherence through personalized deals and promotions (18). In a revision of apps for IMID patients, only 1 of the 243 apps analyzed has a gamification module (10).

Finally, our results revealed a high degree of satisfaction with the aspects assessed. Those best rated were the ability to communicate with healthcare professionals, ease of app use, and reminders to administer medication. Patient satisfaction with this type of app is generally high (23, 31). Fifty-five patients with Crohn’s disease were satisfied (median VAS score 8) with an app for monitoring their intravenous biological treatment (31). The mean satisfaction with the Sanoia^®^ platform was very high, although around a quarter of the patients never accessed the platform (12). The retention rate for eMidCare was high. We found no published studies on the usability of apps for IMID patients; however, 90-day retention has been reported to be 34% and annual retention 16% (32). The success of eMidCare was probably due to its *ad hoc* development based on patients’ needs and the possibility of communication with health professionals. Support from health professionals is key to increasing longer-term engagement with these tools. Besides, apps that involve the active participation of patients by data registration, messages, or gamification can improve usability. The possibility for healthcare professionals to adapt the contents of the app through the CMS for ease of reproducibility and acceptance by patients

### 4.2 Limitations

While health apps encourage patients to take an active role in the management of their disease, this may lead them to overestimate their role in choosing medical treatment. It is essential that the app adequately detects “alarms” and that healthcare professionals have sufficient control mechanisms (33); hence the importance of the web interface, which enables continuous and real-time monitoring of patients. However, this supervisory role of healthcare professionals could increase their workload. eMidCare has a system for sending automatic emails to healthcare professionals when patients add a new medication, register an AE, or send a message. Consequently, health professionals know when these events occur without having to check the website, thus continuously reducing workload.

The limited size sample of patients diagnosed with IBD and JIA makes it difficult to draw conclusions in these pathologies. However, most treatments for these pathologies are common to other IMIDs. Thus, the information and questions about treatment and the adverse events that patients suffer are very similar.

We evaluated the use of our app through a prospective observational study. The descriptive evaluation of the results in our patients and the absence of a comparator group do not allow us to draw solid conclusions as to whether eMidCare improves certain aspects such as medication safety or adherence. Some review studies on the use of apps in IMID patients conclude that evidence on their impact is still lacking and that clinical trials with clearly predefined outcomes should be performed in a large cohort (33). However, the rapid advancement of mHealth makes this problematic owing to changes in technologies. The positive results we obtained serve as an incentive to continue using eMidCare with our patients, and we will continue to make improvements and carry out evaluations of this system.

### 4.3 Conclusions

We developed an app, eMidCare^®^, which enables remote and real-time follow-up of and communication with IMID patients receiving biological therapies. This new tool reminds patients to take their medication, enables them to record AEs, and helps them communicate with healthcare professionals. Approximately one-third of the patients registered the administration of the biological therapies and registered at least 1 AE. However, the most used and most satisfactory functionality was communication with health professionals. Patient satisfaction and retention were very high.

## Data availability statement

The raw data supporting the conclusions of this article will be made available by the authors, without undue reservation.

## Ethics statement

The study was reviewed and approved by the Clinical Research Ethics Committee of Gregorio Marañón University Hospital (code MSS-APP-2019). All patients signed written informed consent.

## Author contributions

RR-J wrote the first draft. RR-J, VE-V, AH, and MS defined the research question and objectives. RR-J, VE-V, EC-d-V, AA-L, EL-M, CG, OB, LM, CL-R, and AH defined the app’s functionalities. RR-J, VE-V, EC-d-V, AA-L, EL-M, BS-F, and PR-B included the patients and followed them up. RR-J, VE-V, AH, and MS were responsible for the research activity plan and its execution. All authors reviewed and approved the manuscript.

## Funding

Novartis sponsored the technological development of the app. Novartis did not participate in the conception of the app, the inclusion and follow-up of patients, or the evaluation of results.

## Conflict of interest

The authors declare that the research was conducted in the absence of any commercial or financial relationships that could be construed as a potential conflict of interest.

## Publisher’s note

All claims expressed in this article are solely those of the authors and do not necessarily represent those of their affiliated organizations, or those of the publisher, the editors and the reviewers. Any product that may be evaluated in this article, or claim that may be made by its manufacturer, is not guaranteed or endorsed by the publisher.
