# Supplementary material for: Design and implementation of a mobile app for the pharmacotherapeutic follow-up of patients diagnosed with immune-mediated inflammatory diseases: eMidCare
Source: Front Immunol. 2022 Jul 28;13:915578. doi: 10.3389/fimmu.2022.915578 (PMC9367980; doi:10.3389/fimmu.2022.915578)
Supplement: Supplementary file 1 [file DataSheet_1.doc]

**SUPPLEMENTAL MATERIAL**

**1. eMidCare app satisfaction survey**

Please, select the number that best describes your opinion for each of the following statements, with 1 being "strongly disagree" and 10 being "strongly agree".

All information received is strictly confidential and anonymous, and will be of great use to us.

| 1. Is the app helpful for you? | 1 | 2 | 3 | 4 | 5 | 6 | 7 | 8 | 9 | 10 |
| --- | --- | --- | --- | --- | --- | --- | --- | --- | --- | --- |
| 2. Do you think the app is easy to use? | 1 | 2 | 3 | 4 | 5 | 6 | 7 | 8 | 9 | 10 |
| 3. Do you think the app helps you to manage your appointments with healthcare professionals? | 1 | 2 | 3 | 4 | 5 | 6 | 7 | 8 | 9 | 10 |
| 4. Do you think the app helps you to remember to administer or take your medication? | 1 | 2 | 3 | 4 | 5 | 6 | 7 | 8 | 9 | 10 |
| 5. Do you think the app makes contact with your pharmacist easier for you? | 1 | 2 | 3 | 4 | 5 | 6 | 7 | 8 | 9 | 10 |
| 6. Do you find useful to complete the quality of life questionnaires through the app? | 1 | 2 | 3 | 4 | 5 | 6 | 7 | 8 | 9 | 10 |
| 7. Do you think the app helps you to manage the adverse events of the treatment? | 1 | 2 | 3 | 4 | 5 | 6 | 7 | 8 | 9 | 10 |
| 8. Do you think the app helps you to get information about your treatment, symptoms, or lifestyle? | 1 | 2 | 3 | 4 | 5 | 6 | 7 | 8 | 9 | 10 |
| 9. Would you recommend the app to someone else in your situation? | 1 | 2 | 3 | 4 | 5 | 6 | 7 | 8 | 9 | 10 |

10. Which characteristic of the app do you like the most or find more useful? (you can choose more than one answer; select as many as you consider):

(1) Easy to use

(2) It gives me more autonomy and security

(3) To able to communicate with the pharmacist from any location

(4) To able to communicate with the pharmacist at any time

(5) The immediacy of the response

(6) To be able to manage the adverse events of the treatment from home

(7) To be able to register the quality of life questionnaires in the app instead of on paper

(8) Obtain information about the treatment and the disease

(9) To have the treatment recorded and to be alerted when I take the medication

(10) Check the compatibility of the medicines I am taking

(11) Other (tell us other positive aspects of the app):

11. Which characteristic of the app do you like least or find least useful? (you can choose more than one answer; select as many as you consider):

(1) Difficult to use

(2) It does not give me more autonomy or security

(3) To be able to communicate with the pharmacist from any location

(4) To be able to communicate with the pharmacist at any time

(5) The immediacy of the response

(6) To be able to manage the adverse events of the treatment from home

(7) To be able to register the quality of life questionnaires in the app instead of on paper

(8) Obtain information about the treatment and the disease

(9) To have the treatment recorded and to be alerted when I take the medication

(10) Check the compatibility of the medicines I am taking

(11) Other (tell us other negative aspects of the app):

12. Please, rate your overall satisfaction with the app from 1 to 10:

13. Help us to improve, comment on what aspects of the application you would improve:

**2. Decision tree of adverse events.**

| **Adverse event** | **Question asked by the app** | **Answer by the patient** | **CTCAE severity** | **Recommendation by the app** |
| --- | --- | --- | --- | --- |
| **Diarrhea** | How many bowel movements have you made in the last 24 hours? | 1 | 1 | *The app displays a brochure with general recommendations on the prevention and management of diarrhea* |
|  |  | 2 |  |  |
|  |  | 3 |  |  |
|  |  | 4 | 2 | - If you have fever, poor general condition, or cannot tolerate food or fluids, or you have black or stools with pus or blood, **go to the emergency department**.  - If you do NOT have any of the above symptoms and you are taking loperamide, **contact your general practitioner or hospital specialist.**  - If you do NOT have any of the symptoms listed above and you are NOT taking loperamide, **check the clinical information you have been given to decide if you can take 1 capsule of loperamide after each bowel movement, up to a maximum of 8 capsules per day. If you do not improve within 24 hours, contact your general practitioner or hospital specialist**. |
|  |  | 5 |  |  |
|  |  | 6 |  |  |
|  |  | 7 | ≥3 | - If you have fever, poor general condition, or cannot tolerate food or fluids, or you have black or stools with pus or blood, **go to the emergency department**.  - If you do NOT have any of the above symptoms, **contact your specialist physician at the hospital. If this is not possible, go to the emergency department.** |
|  |  | 8 |  |  |
|  |  | 9 |  |  |
|  |  | 10 or more |  |  |
| **Vomiting** | How many vomiting have you had in the last 24 hours? | 1 | 1 | *The app displays a brochure with general recommendations on the prevention and management of vomiting* |
|  |  | 2 |  |  |
|  |  | 3 | 2 | - If you have fever, poor general condition, or cannot tolerate food or fluids**,** **go to the emergency department**.  - If you do NOT have any of the above symptoms, **contact your hospital pharmacist, general practitioner or hospital nurse.** |
|  |  | 4 |  |  |
|  |  | 5 |  |  |
|  |  | 6 | ≥3 | Go to the emergency department. |
|  |  | 7 |  |  |
|  |  | 8 |  |  |
|  |  | 9 |  |  |
|  |  | 10 or more |  |  |
| **Nausea** | Please, select the closest match: | I have less appetite, although I eat more or less the same as usual | 1 | *The app displays a brochure with general recommendations on the prevention and management of nausea* |
|  |  | I eat less since I started the new treatment, although I have hardly lost any weight | 2 | - If you have fever, poor general condition, or cannot tolerate food or fluids**,** **go to the emergency department**.  - If you do NOT have any of the above symptoms, **contact your hospital pharmacist, general practitioner or hospital nurse.** |
|  |  | I eat less since I started the new treatment and have lost weight lately | 3 | - If you have fever, poor general condition, or cannot tolerate food or fluids**,** **go to the emergency department**.  - If you do NOT have any of the above symptoms, **contact your specialist physician at the hospital. If this is not possible, go to the emergency department.** |
| **Fever** | If you have a fever, please register your temperature: ____, and , select the closest match: | I have had a fever for less than 24 hours and I feel fine | -38-39º C= 1  -39-40º C= 2  ->40º C less than 24h= 3  ->40º C more than 24h= 4 | Check the information you received to decide whether you can take paracetamol 500 mg every 8 hours. |
|  |  | I have had a fever for less than 24 hours and I feel fine  I have fever for more than 24 hour |  | - If you suffer any of the following symptoms: diarrhea, vomiting, skin rash, difficulty breathing, cough, confusion or low level of consciousness, dizziness, low blood pressure, or new onset abdominal or chest pain, **go to the emergency department**.  - If you do NOT have any of the above symptoms, **contact your general practitioner, hospital pharmacist, specialist physician or hospital nurse.** |
| **Fatigue** | Please, select the closest match: | My fatigue relieves by rest | 1 | *The app displays a brochure with general recommendations on the prevention and management of fatigue* |
|  |  | My fatigue doesn`t relieve by rest (I find it difficult to do some activities, such as walking or shopping). | 2 | Contact your hospital pharmacist or general practitioner, and discuss it with your specialist physician at the next hospital visit. |
|  |  | Due to fatigue, I have significant difficulty with some self-care activities (such as showering or dressing) or significant shortness of breath that does not relieve by rest. | 3 | Go to the emergency department. |
| **Reaction at the injection site** | Please, select the closest match: | I feel tenderness with or without redness, itching or rash when injected. | 1 | *The app displays a brochure with general recommendations on the prevention and management of reaction at the injection site* |
|  |  | I have pain, inflammation, or swelling when I get the injection. | 2 | Go to the hospital nurse. |
|  |  | I have significant skin damage, such as ulcers. | ≥3 | Go to the emergency department. |
| **Headache** | Please, select the closest match: | I have mild pain, which does not limit my normal daily activities. | 1 | *The app displays a brochure with general recommendations on the prevention and management of headache.* |
|  |  | I have moderate pain, which interferes with my usual daily life activities, such as working, walking, talking on the phone, etc. | 2 | Check the information you received to decide whether you can take painkillers, such as paracetamol. If the pain still does not relieve, contact your hospital pharmacist or general practitioner, and discuss it with your specialist physician at the next hospital visit. |
|  |  | I have severe pain, which interferes with my self-care activities (such as showering or dressing | 3 | Go to the emergency department. |
| **Other** | Please, write your adverse event _______, and select the closest match: | This adverse event does not limits my activities of daily living, such as walking or shopping. | 1 | If this adverse event continues or increases, **contact your hospital pharmacist or general practitioner.** |
|  |  | This adverse event limits my activities of daily living, such as walking or shopping. | 2 | Contact your hospital pharmacist or general practitioner. |
|  |  | This adverse event limits my self-care activities, such as showering and dressing. | ≥3 | Contact your hospital pharmacist or specialist physician at the hospital. |

### CTCAE: Common Terminology Criteria for Adverse Events.
